# Implication of the cause of differences in 3D structures of proteins with high sequence identity based on analyses of amino acid sequences and 3D structures

**DOI:** 10.1186/1756-0500-7-654

**Published:** 2014-09-18

**Authors:** Masanari Matsuoka, Masatake Sugita, Takeshi Kikuchi

**Affiliations:** Department of Bioinformatics, College of Life Sciences, Ritsumeikan University, 1-1-1 Nojihigashi, Kusatsu, Shiga, Japan; Japan Society for the Promotion of Science (JSPS) Research Fellow DC2, Tokyo, Japan

**Keywords:** Artificial homologues, Chameleon sequence, Sequence analysis, Inter-residue average distance statistics, Conservation analysis, Sequence tendency, IgG binding protein, Protein A, Protein G

## Abstract

**Background:**

Proteins that share a high sequence homology while exhibiting drastically different 3D structures are investigated in this study. Recently, artificial proteins related to the sequences of the GA and IgG binding GB domains of human serum albumin have been designed. These artificial proteins, referred to as GA and GB, share 98% amino acid sequence identity but exhibit different 3D structures, namely, a 3α bundle versus a 4β + α structure. Discriminating between their 3D structures based on their amino acid sequences is a very difficult problem. In the present work, in addition to using bioinformatics techniques, an analysis based on inter-residue average distance statistics is used to address this problem.

**Results:**

It was hard to distinguish which structure a given sequence would take only with the results of ordinary analyses like BLAST and conservation analyses. However, in addition to these analyses, with the analysis based on the inter-residue average distance statistics and our sequence tendency analysis, we could infer which part would play an important role in its structural formation.

**Conclusions:**

The results suggest possible determinants of the different 3D structures for sequences with high sequence identity. The possibility of discriminating between the 3D structures based on the given sequences is also discussed.

**Electronic supplementary material:**

The online version of this article (doi:10.1186/1756-0500-7-654) contains supplementary material, which is available to authorized users.

## Background

**Figure 1:**
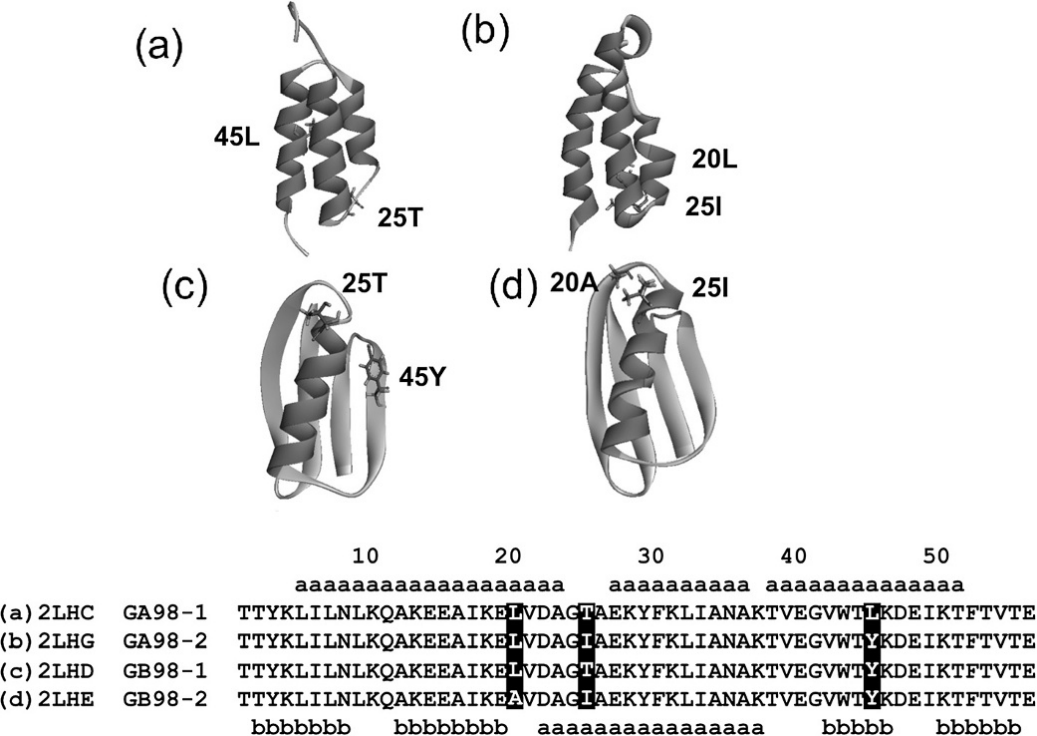
**Ribbon representations of the 3D structures of 2LHC (a), 2LHG (b), 2LHD (c) and 2LHE (d) with their amino acid sequences.** A segment with dark gray denotes an α helix and one with light gray denotes a β strand. The meanings of the symbols **"a"** and **"b"** are same as that in Figure 11. The differences between the sequences are highlighted and shown in stick in the figure above.

In molecular bioinformatics, elucidating how a protein folds into its native structure is a significant unsolved problem that is related to the modelling and design of new protein 3D structures. To address this problem, we have to understand the relationship between the amino acid sequence and the 3D structure of a protein. How the information regarding a protein's folding is coded in its sequence is not yet fully understood. It is well known that the 3D structures of two proteins are similar if the sequence identity is high. In particular, it is generally believed that the topologies of two proteins are usually similar to each other if their sequences share more than about 30% identity [1]. However, recently some sequences of proteins that do not follow this empirical rule have been artificially made. Using the phage display technique to introduce mutations, He et al. [2] succeeded in designing two sequences from those of GA and GB proteins with about 60% identity but different 3D structures, that is, a 3α-helix bundle or a 4β-sheet + α-helix structure. In 2012 He et al. [3] reported two related sequences that differ by only one amino acid in 56 residues yet exhibit the different 3D structures (i.e., 3α bundle or a 4β + α structure - the difference exists only at the 20th or 45th residue; see Figure 1 in detail). He and coworkers analyzed the NMR structures of these proteins in detail with the predicted structures by means of a protein 3D structure prediction technique, ROSETTA, with the help of NMR chemical shift data [3, 4]. Having two proteins whose sequences differ by only one amino acid and yet have different structures makes it difficult to predict which structure is assumed for each of the two sequences based only on sequence information from standard sequence analyses. Discriminating between two alternative structures with a very high sequence identity on the basis of energetics simulations is also considered difficult [5].

In this study, we consider how these amino acid sequences can be decoded to discriminate between their 3D structures and to what degree this is possible at the present stage.

For this purpose, we examine whether the inter-residue average distance statistics can be used to extract new information on 3D structures from these sequences in addition to the information gained from the standard sequence analysis techniques. In a series of studies, we have applied an analysis method based on inter-residue average distance statistics to predict the location of structural domains [6], compact regions during the folding of fatty acid binding proteins [7], globin fold proteins [8], c-type lysozyme proteins [9] and β-sandwich proteins [10]. This technique has also been used to analyze the GA and GB proteins' related sequences, which are 60% identical to each other, and the sequential properties that result in the exhibition of either structure have been determined [11]. In the present study, we focus on the sequences of the GA and GB proteins, which differ in only one amino acid but exhibit different 3D structures, that is, 3α or 4β +α folds and related sequences. The signatures of a sequence that characterize the differences in folding and possible ways to discriminate between the 3D structures are discussed.

## Results

### BLAST search

When the PDB was searched for protein sequences homologous with that of [PDB:2FS1] (GA), the number of hit sequences was 11 after identical sequences and sequences with less than 28 residues were excluded. When the [PDB:1PGA] (GB) sequence was used as a query, the number of the hit sequences was 42 after sequences with the same criteria were excluded.

Out of the 11 sequences found using the 2FS1 sequence as a query, seven sequences have 3D structures of 3α, including [PDB:2LHC] (GA98-1) and [PDB:2LHG] (GA98-2); four sequences show the 4β + α fold, including [PDB:2LHD] (GB98-1) and [PDB:2LHE] (GB98-2); and no sequences have other structures.

The majority of the hit sequences found with the [PDB:1PGA] (GA) query exhibit the 4β + α structures, that is, the 3D structures of 34 sequences show a 4β + α fold, including [PDB:2LHD] (GB98-1) and [PDB:2LHE] (GB98-2). The 3D structures of four sequences are 3α, including [PDB:2LHC] (GA98-1) and [PDB:2LHG] (GA98-2), and the rest of the hit sequences have other structures. These results are summarized in Table 1. Thus, BLAST tends to distinguish the 4β + α fold from other folds, but the results are still contaminated by some 3α structures. As a result, it is difficult to distinguish between 3α and 4β + α structures based on only sequence identity, especially for the case where the two sequences share a very high identity, such as 98%. The wrong hit cases in the BLAST searches always show e-values of less than 10^-6^.

**Table 1:** **Breakdown of the hit results from BLAST searches with the following queries**

3D structure of hit sequences	Query	
	2FS1	1PGA
3α	7	4
4β + α	4	34
Other	0	4
Total	11	42

### F-value analysis

**Figure 2:**
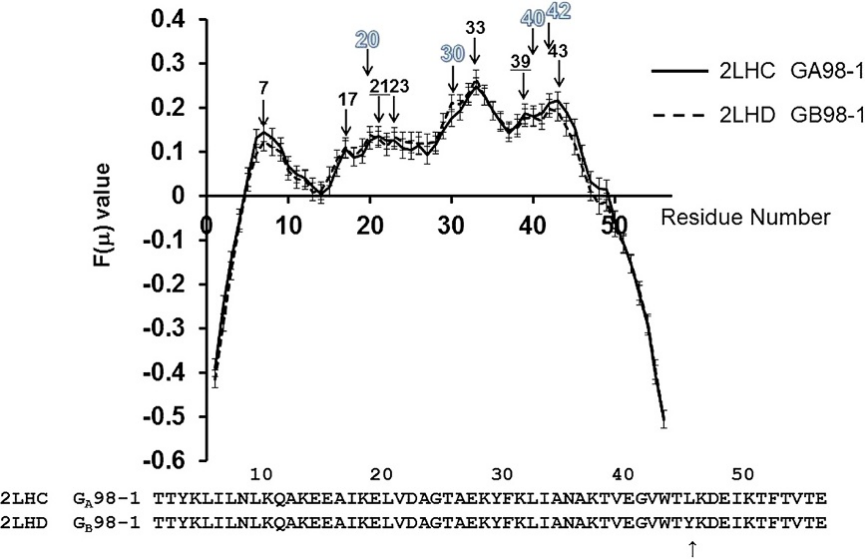
**Plots of F(μ) values for 2LHC and 2LHD with error bars.** An arrow with a numeral denotes the location of a peak of a plot. The numeral indicates the position of the residue at a peak. A black numeral, an underlined black numeral and an outlined numeral mean a peak observed in both 2LHC (GA98-1) and 2LHD (GB98-1), a peak observed in only 2LHC, and a peak observed in only 2LHD, respectively.

**Figure 3:**
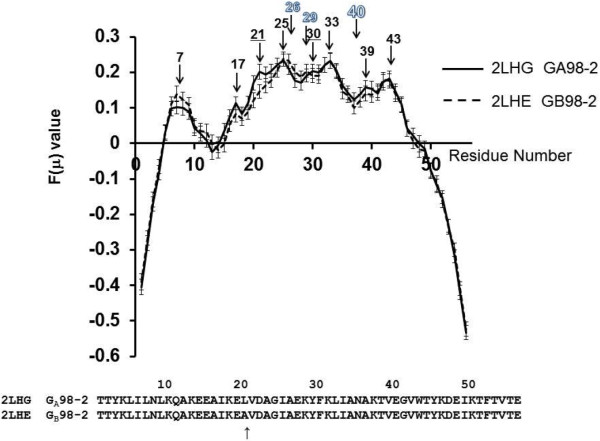
**Plots of F(μ) values for 2LHG and 2LHE with error bars.** An arrow with a numeral denotes the location of a peak of a plot. The numeral indicates the position of the residue at a peak. A black numeral, an underlined black numeral and an outlined numeral mean a peak observed in both 2LHG (GA98-2) and 2LHE (GB98-2), a peak observed in only 2LHG and a peak observed in only 2LHE, respectively.

The results of F-value calculations are presented in Figures 2 and 3. The results for [PDB:2LHC] (GA98-1) and [PDB:2LHD] (GB98-1) are shown in Figure 2, and the results for [PDB:2LHG] (GA98-2) and [PDB:2LHE] (GB98-2) are shown in Figure 3.

**Figure 4:**
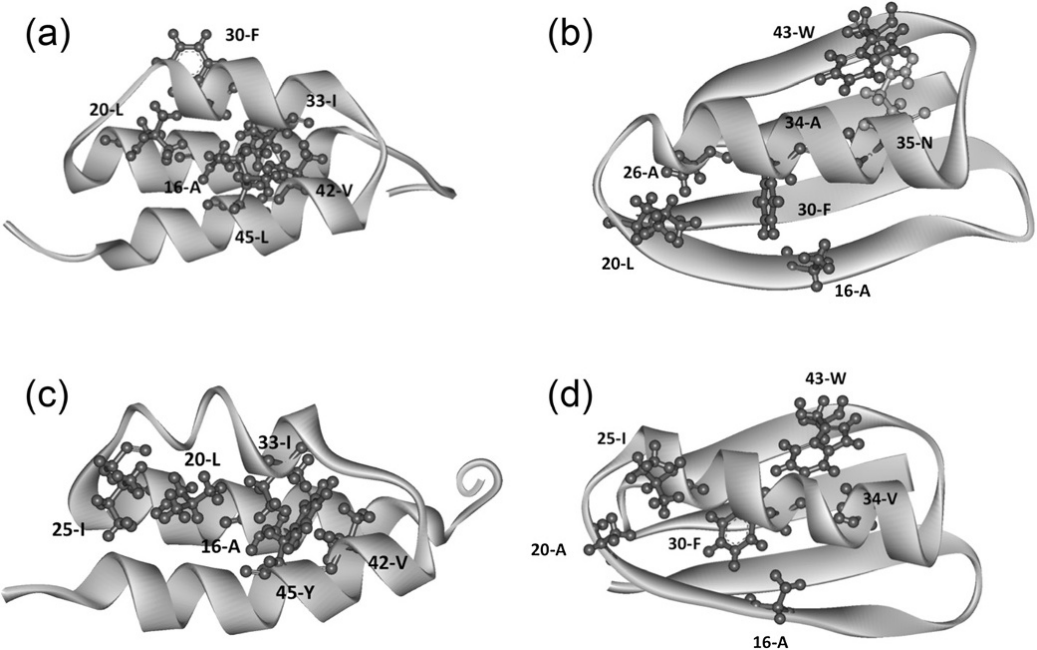
**Visuallization of hydrophobic packings. (a)** The packing hydrophobic residues formed by residues near the peaks of the F-value plot for 2LHC (GA98-1). The packing residues are 16-A, 20-L, 30-F, 33-I, 42-V and 45-L. **(b)** The hydrophobic contacts formed by residues near the peaks of the F-value plot for 2LHD (GB98-1). The pairwise contacts are formed by 16-Ala and 30-Phe, 20-Leu and 26-Ala as well as by 34-Ala and 43-Trp. 35-Asn, which forms a contact with 43-Trp in Gō model simulations, is indicated by light gray. **(c)** The packing hydrophobic residues formed by residues near the peaks of the F-value plot for 2LHG (GA98-2). The packing residues are 16-A, 20-L,25-I, 33-I, 42-V and 45YL. **(d)** The hydrophobic contacts formed by residues near the peaks of the F-value plot for 2LHE (GB98-2). The pairwise contacts are formed by 16-Ala and 30-Phe, 20-Leu and 25-Ile as well as by 34-Ala and 43-Trp.

Clearly, F-value analysis cannot detect any sharp differences between the GA98 and GB98. The plot of F-values for [PDB:2LHC] (GA98-1) in Figure 2 shows peaks at the 7th, 17th, 21st, 23rd, 33rd, 39th, and 43rd residues. For [PDB:2LHD] (GB98-1), the peaks are observed at 7th, 17th, 20th, 23rd, 30th (shoulder), 33rd, 40th and 42nd residues. The detailed observation of the 3D structure of [PDB:2LHC] (GA98-1) reveals that 16-Ala, 20-Leu, 30-Phe, 33-Ile, 42-Val, and 45-Leu form hydrophobic packing as seen in Figure 4(a). In particular, the significance of the packing of 33-Ile and 45-Leu is also noted by He at al. [3] That is, hydrophobic residues at the peaks of the F-value plot form hydrophobic packing in the actual 3D structure. Here, packing is defined as the case where one of the heavy atoms in the i-th residue locates within 5 Å of any heavy atoms in the j-th residue in the native structure. For [PDB:2LHD] (GB98-1), pairwise hydrophobic packing is formed by 16-Ala and 30-Phe, 20-Leu and 26-Ala, as well as 34-Ala and 43-Trp. In other words, residues near the peaks of the F-value plot (with at most a ± 3 residue difference as seen in the case of 26-Ala in [PDB:2LHD] (GB98-1) take part in hydrophobic contacts as presented in Figure 4(b). In both the cases, for [PDB:2LHC] (GA98-1) and [PDB:2LHD] (GB98-1), the hydrophobic packing is formed by the residues near the peaks of the F-value plots, but the residues involved in the hydrophobic packing are slightly different between [PDB:2LHC] (GA98-1) and [PDB:2LHD] (GB98-1). The results suggest that 45-Y does not participate in contact formations with any residue near the peaks of the F-value plot in [PDB:2LHD] (GB98-1).

In the same way, for [PDB:2LHG] (GA98-2), the peaks of the F-value plot also appear at 7th, 17th, 21st, 25th, 30th (shoulder), 33rd, 39th and 43rd residues. For [PDB:2LHE] (GB98-2), the peaks are observed at 7th, 17th, 25th, 26th, 29th, 33rd, 39th (shoulder) and 43rd residues. In comparison with the cases of [PDB:2LHC] (GA98-1) and [PDB:2LHD] (GB98-1), the peaks around 25 are remarkable for [PDB:2LHG] (GA98-2) and [PDB:2LHE] (GB98-2), reflecting the mutation at the 25th residue from Thr to Ile. The 3D structure of [PDB:2LHG] (GA98-2) shows the hydrophobic packing by 16-Ala, 20-Leu, 25-Ile, 33-Ile, 42-Val and 45-Tyr (Figure 4(c)). In the 3D structure of [PDB:2LHE] (GB98-2), hydrophobic contacts are formed by 16-Ala and 30-Phe, 20-Ala and 25-Ile, as well as 34-Ala and 43-Trp in Figure 4(d).

Similar phenomena, such as the hydrophobic residues around the peaks of an F-value plot, correspond to the residues forming hydrophobic contacts and can be observed in several proteins such as β-sandwich proteins [10], 60% homologous proteins related to GA and GB proteins [11], ferredoxin-like proteins [12], and so on.

Comparing the 3D structures of these four proteins as shown in Figures 4(a)-(d), one finds that in the 3α structures, the formation of the hydrophobic cluster appears to be the driving force of 3D structure formation. On the other hand, formation of the individual hydrophobic contact by a pair of hydrophobic residues with the central helix reveals significant 4β + α structures. In the case of [PDB:2LHC] (GA98-1) and [PDB:2LHD] (GB98-1), the difference is just the 45th residue, that is, Leu and Tyr. In [PDB:2LHC] (GA98-1), the 45th Leu is actively involved in the hydrophobic cluster. In contrast, the corresponding 45-Tyr does not participate in a hydrophobic contact with the residues near a peak in [PDB:2LHD] (GB98-1).

We performed the same analyses for several other sequences of GA/GB related proteins. Similar results were obtained and presented in our [Additional file 1].

### Local sequence tendencies

**Figure 5:**
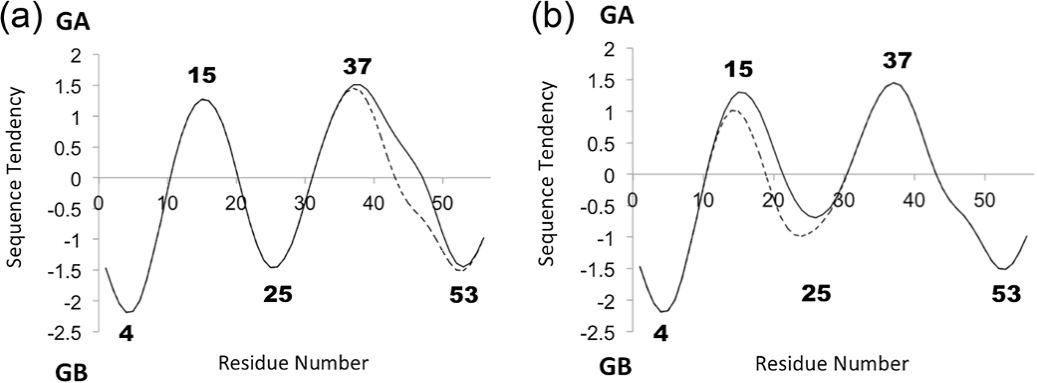
**Sequence tendencies. (a)** The solid or dashed line corresponds to [PDB:2LHC] (GA98-1) or [PDB:2LHD] (GB98-1), respectively. **(b)** The solid or dashed line corresponds to [PDB:2LHG] (GA98-2) or [PDB:2LHE] (GB98-2), respectively. While the x-axis denotes residue number, the y-axis denotes the relative similarity to 2FS1/1PGA. Positive or negative large value means the local sequence has high similarity to [PDB:2FS1] (GA) or [PDB:1PGA] (GB), respectively. The bold numbers represent the residue numbers at peaks or valleys of the plot. How we get the sequence tendencies is described in the Material and Methods section.

Figure 5 shows the result of sequence tendency calculations. While a positive large value means the corresponding local sequence is mainly descended from [PDB:2FS1] (GA), a negative large value means its sequence is mainly from [PDB:1PGA] (GB). Solid or dashed line denotes the sequence tendency for GA98-1 or GB98-1, respectively. According to this figure, residues around residues 4, 25 and 53 mainly come from the sequence of [PDB:1PGA] and residues around residues 15 and 37 come from that of [PDB:2FS1].

The values of the sequence tendency of the residues around 10, 20, 33, and 45 are almost 0 suggesting the partial sequences around these residues are quite unbiased. However, it is quite interesting that the remarkable difference between solid and dashed lines is also observed between residues in the range 37–53, that is, around 0 values of the sequence tendency. The difference leads the local sequence of GA98-1 to be similar to [PDB:2FS1], while the local sequence of GB98-1 to be similar to [PDB:1PGA]. The F-value results show peaks around the same place. Here, other remarkable peaks and valleys based on both the F-values and sequence tendencies are as follows: Besides the remarkable difference around residue 25 of the two F-value plots shown in Figures 2 and 3, the main peaks are observed commonly in both figures, that is, around residue 7, (17), 33, (39) and 43 (parentheses are used for insignificant peaks). According to Figure 5 of sequence tendencies, one peak locates around residue 15 and another peak locates around 35. Similarly, valleys locate around residue 4, 25 or 53, while the difference is observed around residue 45 or 20.

**Figure 6:**
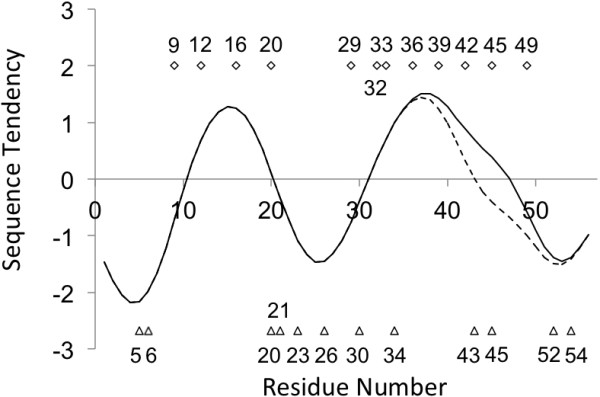
**Distribution of conserved hydrophobic residues with local sequence tendencies.** The solid or dashed line corresponds to the sequence tendency of GA98-1 or GB98-1, respectively. The squares above the sequence tendency plot denote the conserved hydrophobic residues of 2FS1 and its homologues (shown in Figure 8), while the triangles below the tendency plot denote these of 1PGA (shown in Figure 9).

It is also notable that a sequence tendency comes and goes between positive and negative values about every 10–15 residues. Interestingly, conserved hydrophobic residues (described in the next "Sequence Alignments" section) distributes where these positive value peaks or negative value valleys exist shown in Figure 6. This may be valuable information when one wants to create another new chimera protein. See the [Additional file 1] for other homologous pairs.

### Sequence alignments

**Figure 7:**
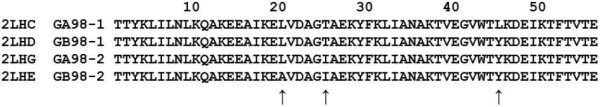
**Multiple alignment of 2LHC, 2LHD, 2LHG and 2LHE.** The sites beside those marked by arrows are perfectly conserved.

The sequence alignments of [PDB:2LHC] (GA98-1), [PDB:2LHD] (GB98-1), [PDB:2LHG] (GA98-2) and [PDB:2LHE] (GB98-2) are shown in Figure 7. As we mentioned in Background, the differences are just the 20th, 25th and 45th residues. Therefore, the N- and C-terminal ends do not seem to be the most important determinants of the 3D structures, because indeed the N-terminus 19 and C-terminus 11 residues are exactly the same for four sequences.

This means that the N- and C-terminal ends do not play a main role in the 3D structure formation in the very early stages of folding, although these parts are biased to one of the two original proteins according to Figure 5.

**Figure 8:**
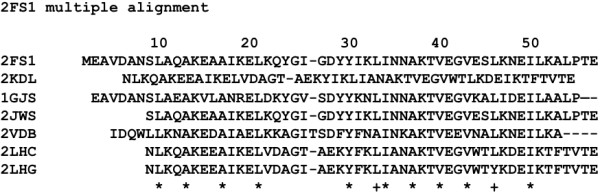
**Multiple alignment of sequences of 2FS1 and related proteins hit by BLAST.** A site marked by "*" is perfectly conserved and that marked by "+" is 85% conserved.

The alignment of the hit sequences by BLAST with the [PDB:2FS1] sequence as a query is shown in Figure 8. The perfectly conserved hydrophobic residues are labelled with *, and the case in which just one residue is mutated by another hydrophobic residue is labelled with +. One mutation at a given site means an 85% conservation for the present case. This is true for 10-Ala, 12-Ala, 16-Ala, 20-Leu, 29-Tyr, 33-Ile, 36-Ala, 39-Val, 42-Val and 49-Ile. These conserved residues correspond well to the residues forming the hydrophobic cluster in the 3α structure. The residues significant for native structure formation are thought to be evolutionally conserved [13, 14].

**Figure 9:**
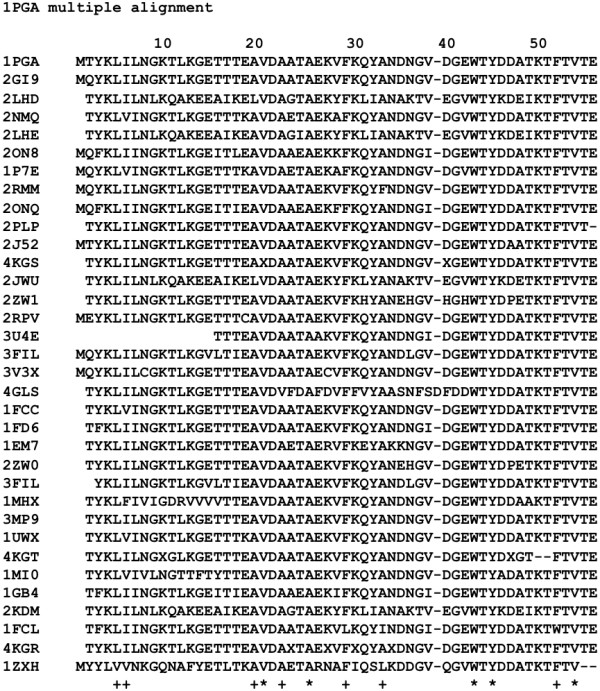
**Multiple alignment of sequences of 1PGA and related proteins hit by BLAST.** A site marked by "*" is perfectly conserved and that marked by "+" is 85% conserved.

In the alignment made by the hit residues with the [PDB:1PGA] sequence as a query, the perfectly conserved hydrophobic residues are 21-Val, 26-Ala, 43-Trp, 45-Tyr, and 54-Val as seen in Figure 9. A residue labelled with + indicates that less than 4 residues have been mutated by other hydrophobic residues (corresponding to 85% conservation). More than 85% conserved hydrophobic residues are also observed at positions 5, 6, 20, 23, 26, 30, 34, and 52. These situations are summarized in Table 2. The packing residues corresponding to values around the peaks of F-value plots are conserved hydrophobic residues. Similar occurrences have been observed in ferredoxin-like proteins [12].

**Table 2:** **Correspondence between conserved hydrophobic residues in multiple alignments and packing residues in 3D structure**

3α structure	
Conserved hydrophobic residues	9-L, 12-A, 16-A, 20-L, 29-Y, 32-L(+), 33-I, 36-A, 39-V, 42-V, 45-L(+), 49-I
Packing residues in 2LHC	16-A, 20-L, 30-F, 33-I, 42-V, 45-L
Packing residues in 2LHG	16-A, 20-L, 25-I, 33-I, 42-V, 45-L
4β + α structure	
Conserved hydrophobic residues	5-L(+), 20-A(+), 21-V, 23-A(+), 26-A, 30-F(+), 34-A(+), 43-W, 45-Y, 52-F(+), 54-V
Packing residues in 2LHD	16-A, 20-L, 26-A, 30-F, 34-A, 43-W
Packing residues in 2LHE	16-A, 20-L, 25-I, 34-A, 43-W

"+" denotes a site in multiple alignment with more than 85% conservation.

### Gō-model simulations

**Figure 10:**
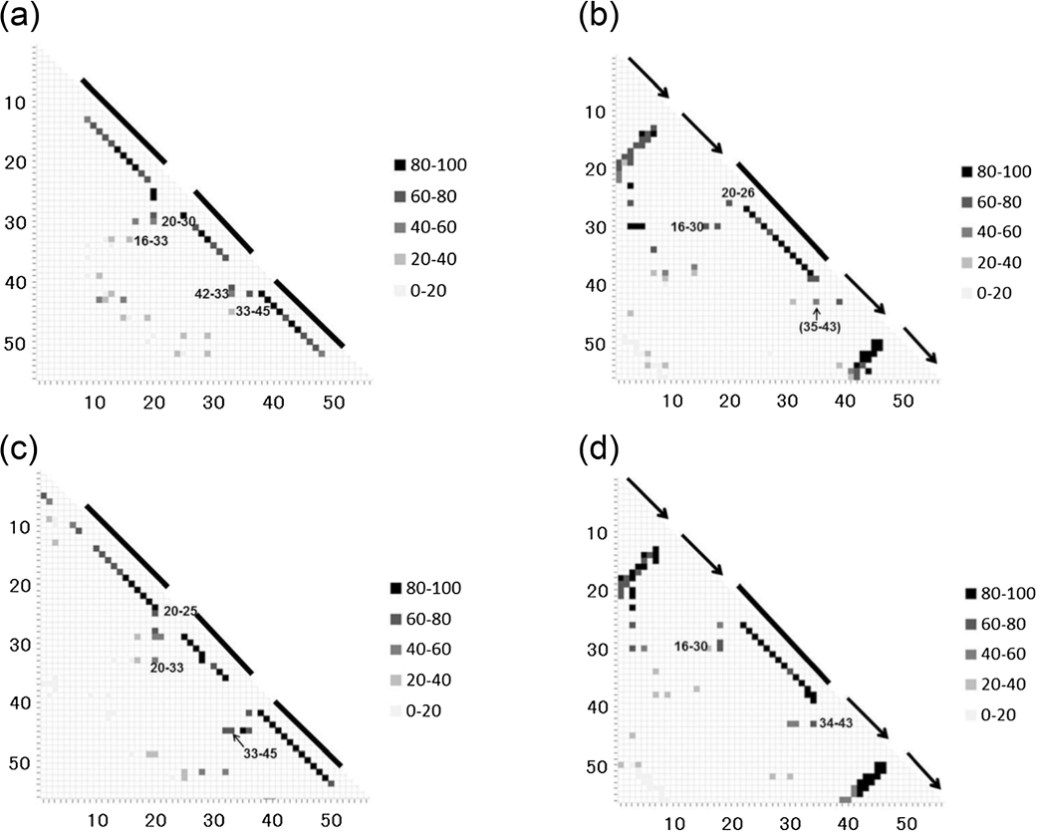
**Contact map constructed from the conformation ensemble simulated by the present Gō model at around the transition state of folding. (a)** 2LHC, **(b)** 2LHD, **(c)** 2LHG, and **(d)** 2LHE. A darker spot indicates the high occurrence of conformations with a contact corresponding to the spot. The numbers, for example "80-100", on the right side of the figure are the number of conformations during a simulation near the transition state. A black bar denotes the location of an α helix, and a black arrow indicates the location of a β strand.

Figure 10(a) shows a contact map constructed from the conformational ensemble simulated by the present Gō model close to the transition state of folding with Q = 0.52 for [PDB:2LHC] (GA98-1).

A darker spot represents a high occurrence of conformations with a contact corresponding to the spot. Due to the specific nature of the present Gō model, α helices form at a very early stage of folding. Relatively high frequencies of contact formations by 16-Ala and 33-Phe, 20-Leu and 30-Phe, 33-Ile and 42-Val as well as 33-Ile and 45-Leu can be observed in this figure, as expected from the results above. A contact map showing the contact frequencies at the transition state ensemble of the Gō model simulations for [PDB:2LHD] is presented in Figure 10(b) (Q = 0.62). Again, the β sheets at the N- and C-termini form at the early stages of folding due to the specific nature of the present Gō model. However, as mentioned above, because of the high sequence identity at the N- and C-terminal parts of four proteins, these β sheets do not seem to be the main folding units formed actively in the very early stage of folding in 4β + α proteins. Thus, we focus on the central region of a protein. The hydrophobic packing of 16-Ala and 30-Phe as well as of 20-Leu and 26-Ala are shown on the map. Even though the contact between 34-Ala and 43-Trp is missing in the contact map, the interaction between 35-Asn and 43-Trp, which is a contact very close to that between 34-Ala and 43-Trp, is observed. The packing of 35-Asn and 43-Trp is also presented in Figure 10(b). In the same way, the contact map for [PDB:2LHG] (GA98-2) with Q = 0.42 is shown in Figure 10(c). The high frequency hydrophobic contacts are seen at 16-Ala and 25-Ile, 20-Leu and 33-Ile as well as 33-Ile and 45-Tyr. These observations are consistent with the results from the analyses of the peaks of the F-value plot and the 3D structure. The contact map obtained for [PDB:2LHE] (Q = 0.64) is presented in Figure 10(d). The frequent contacts at 16-Ala and 30-Phe as well at 34-Ala and 43-Trp are observed in the map, and these are two of three hydrophobic contacts derived by the F-value plot analysis.

## Discussion

The present results are summarized as follows.

1. It is plausible that in both structures, 3α and 4β + α folds, a residue located near a peak (within ± 3 residues) of an F-value plot participates in forming hydrophobic contacts. Apparently, which residues in a sequence participate in such contacts determines which structure the protein takes.
2. In addition to (1) above, according to our sequence tendency analysis, the difference exists in the local sequence whose sequence similarity is not biased toward either [PDB:1PGA](GA) or [PDB:2FS1](GB) sequences. This difference makes the unbiased sequence to biased sequence toward either structure. The F-value peak there would emphasize the difference and contribute to the structural determination. This determination leads to the difference in structural formation of N- and C-terminal ends, which are assumed to play a passive role in the folding process.(3) Furthermore, some of the highly conserved residues are located around the peaks in F-value plots in both structures. However, the positions of the conserved residues are slightly different between two structures, as observed in Figures  8 and 9.

From the F-value analyses, conserved hydrophobic residues, and Gō model simulations, the following inference for the folding processes can be made. In the very initial stage of folding, the same sites in the sequences probably contribute to folding in both folds. If the main driving force is hydrophobic packing of these sites, the 3D structure becomes a 3α fold. If the main driving force is relatively short interactions around the helix, the fold becomes a 4β + α fold. In the 3α fold, the conserved hydrophobic residues form a hydrophobic cluster at the early stage of folding, and then the 3α bundle structure forms. In contrast, it seems that some pairs of conserved hydrophobic residues form hydrophobic contacts around the central helix in the 4β + α fold. The results of the present Gō model simulations also suggest the contribution of these conserved hydrophobic residues in forming the different 3D protein structures. The significance of hydrophobic packing for 3α-helix structure has been pointed out by He et al. [2], and the formation of the central α helix in the early stage of folding has been reported by van Gunsteren and coworkers [5].

With information on the sequence tendency (or the distribution of conserved hydrophobic residues) and the location of F-value peaks, one can deduce which structures a given protein may take. For the sequences of Figure 3, the two proteins, [PDB:2LHG] (GA98-2) and [PDB:2LHE](GB98-2), have F-value peaks around residue 25 where the local sequence is biased toward the 4β + α fold (Figure 5(b)). That is, the local sequence around this residue is considered to be the initial folding site from the F-value plot and prefers the 4β + α fold from sequence tendency. As mentioned previously, this residue forms a specific hydrophobic contact with 20-Ala to stabilize βα packing. However, the mutation on residue 20 from Ala to Leu produces the shift of the sequence tendency plot to the 3α fold indicating weakening the tendencies to be 4β + α fold as shown in Figure 5(b). With the help of minor F-value peaks around residues 17 and 21, the sequence of [PDB:2LHG] (GA98-2) forms a 3α fold. On the other hand, residue 33 or 43, which exhibits F-value peaks in all proteins as shown in Figures 2 and 3 and considered to be the initial folding site, has almost zero value in sequence tendency. Therefore they are not considered to bias toward either of the structures. This means that residue 33 forms the initial hydrophobic interactions not leading to either of the 3D structures. However, adding some minor difference may bias the local sequence to either side, and it would be the determining factor. In the current case, 45-Tyr in GB98-1 is mutated to Leu in GA98-1, and this mutation shifts the sequence tendency to 3α structure.

Taken together our results suggest that in the early stage of folding, the local sequence around residue 33 would start to fold without orientation toward some specific structure. However, local sequence around the F-value peaks of residue 43 (on both Figures 2 and 3) or residue 25 (only in Figure 3) have some differences in sequence tendency. This difference would result in the difference in structural formation. In addition to this, because the relationship between the conserved hydrophobic residues and the present Gō-model simulation described in the Results section, it can be inferred that the information on the conserved hydrophobic residues helps us understand which residues are important for folding. It is also notable that the distribution density of the conserved hydrophobic residues seems to be high around the peaks or valleys, as shown in Figure 6.

The question is whether we can discriminate between two folds from sequences with very high sequence identity. In the following, we propose a possible way of discriminating based on our current knowledge. Suppose that we have a sequence that would form one of the two different structures. Next its F-value plot is calculated so the positions of the peaks can be identified. Then the homologous sequences are gathered by BLAST and classified according to their structures. If multiple clusters are identified, the conserved hydrophobic residues and the ancestral sequence for each cluster are obtained. These conserved residues could be used for assuming which fold a given sequence may take; in the present case, the conservation of residues around the peaks of the F-value plot is examined. In a given sequence, the residues corresponding to the high conservation sites in 3α fold sequences and 4β + α fold sequences are considered. If the residue of a high conservation site is not a conserved residue in 3α fold sequences, then its fold should be 4β + α and vice versa. For example, we can make a prediction as follows. If the 20th residue is not Leu, then this fold should be 4β +α, because the 20th residue has been perfectly conserved as Leu so far in the sequences with the 3α fold. If the 45th residue is not Tyr, then the fold should be 3α. If the 26th residue is not Ala, then the fold should be 3α.

In addition to this assumption, one can calculate the sequence tendency with a given sequence and the ancestral sequences, which provides us valuable information about which local sequence is similar to some structure. Comparing the position of F-value peaks with the peaks or valleys of sequence tendency would tell us which structure a given sequence would fold. The hydrophobic conserved residues around F-value peaks tell us which residues play important roles.

We understand that the present study provides just a simple and convenient way to distinguish the 3D structures of very homologous sequences. But we also believe that our method gives clues regarding the folding mechanisms of these kinds of proteins. The fundamentals of the folding properties of these proteins should be investigated through more detailed simulations, taking the characteristics identified by the present study into account. Currently we are continuing our studies along this direction and also planning to provide the present technique in our website.

## Conclusions

It is still a difficult problem to discriminate which 3D structure, 3α or 4β + α, will be assumed among sequences with high sequence identity. But, even at the present stage, focusing on the peaks of the F-value plot combined with the knowledge of conservation residues, the key residues that determine a fold may be identified.

## Methods

### Proteins treated in this study

**Figure 11:**
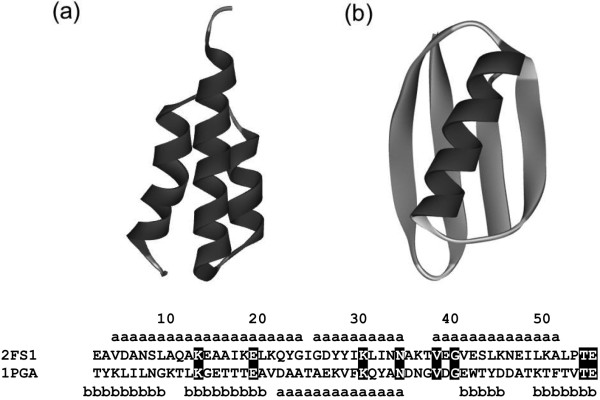
**Ribbon representations of the 3D structures of 2FS1 (a) and 1PGA(b) with their amino acid sequences.** A segment with dark gray denotes an α helix and one with light gray denotes a β strand. A residue with the symbol "a" takes the α-helix conformation and one with "b" takes a β-strand conformation. The definition of the secondary structures in the PDB is used in this study. All identical residues between the two sequences are highlighted.

The human serum albumin (HAS) binding GA domain and the IgG binding GB domain from the *Streptococcus* cell surface protein G are treated in this study. The Protein Data Bank (PDB) codes of these proteins are [PDB:2FS1] (GA) and [PDB:1PGA] (GB), respectively. The 3D structures of these proteins are presented in Figure 11.

We also examine four additional proteins, two of which exhibit 3D structures that are highly similar to that of [PDB:2FS1] (GA), with PDB codes of [PDB:2LHC] (GA98-1) and [PDB:2LHG] (GA98-2). The other two proteins exhibit 3D structures that are highly similar to that of [PDB:1PGA] (GB) with PDB codes of [PDB:2LHD] (GB98-1) and [PDB:2LHE] (GB98-2). Those structures are shown in Figure 1 with their PDB codes.

Throughout the present study, we refer to the proteins by their PDB codes, [PDB:2FS1] and [PDB:1PGA], or simply as GA and GB, respectively. The sequences of these four proteins are highly homologous, as shown in Table 3 and Figure 1. In particular, only one amino acid difference is observed between [PDB:2LHC] (3α) and [PDB:2LHD] (4β + α) and between [PDB:2LHG] (3α) and [PDB:2LHE] (4β + α). A residue difference of only one means a 98% sequence identity. Thus, we designate these proteins as follows: [PDB:2LHC] as GA98-1, [PDB:2LHG] as GA98-2, [PDB:2LHD] as GB98-1 and [PDB:2LHE] as GB98-2. Although we focus on these four proteins, the same analyses in the present work were performed for other sequentially homologous proteins having 3α and 4β + α structures. The results of such proteins are summarized in the [Additional file 1].

**Table 3:** **Sequence identity of a pair of proteins(%)**

2LHD	96		
2LHC	95	98	
2LHG	98	98	96
	2LHE	2LHD	2LHC

### BLAST search

The search for homologous proteins within the PDB was done using BLAST [15] on the following website: http://blast.ncbi.nlm.nih.gov/Blast.cgi. BLOSUM62 was used as a substitution matrix for a sequence alignment. The threshold of the e-value was set as 0.01.

### Simulation of contact formations in a random structure

To analyse the tendency of an amino acid sequence to form contacts between residues, we performed a simulation as follows.

A Cα-beads structure with a bond length of 3.8 Å was used to model a protein structure. Inter-Cα atomic average distance statistics were used to derive a potential for the present simulations [16]. The average distances were calculated using 42 representative proteins with known 3D structures [6]. Considering i-th and j-th residues along a given sequence, a range is defined as the length between two residues along the sequence. That is, the range is defined as M = 1 when 1 ≤ k ≤ 8, where k = |i - j|. The ranges 9 ≤ k ≤ 20, 21 ≤ k ≤ 30, 31 ≤ k ≤ 40 and so on define M = 2, 3, 4 · ··, respectively. An average distance, , for every residue pair in the range M was calculated, where A and B denote the amino acid types.

Let  be an inter-residue effective potential between the i-th and j-th residues. Then,  is expressed as equation (1),
1

where  is the standard deviation and *r*_*ij*_ is the distance between the Cα atoms of the residues i and j in a conformation of a protein during a simulation, and Z is the partition function. Residue types A and B correspond to the residue types of i and j. In the equation, k and T are the Boltzmann constant and temperature, respectively. The constant terms in equation (1) can be regarded as the zero point. In the present study, we set  when . We set *r*_*cut*_ = 1.9 Å and *ε*_*HC*_ = 50 kcal/mol. These values were obtained empirically [16].

Simulations were performed from totally randomized starting conformations. In other words, we conducted a sampling of random structures with the present potential using the standard Metropolis Monte Carlo method. In a Monte Carlo simulation, a dihedral angle, ϕ, and bond angle, θ, of a residue were each varied within - *γπ* ≤ *ϕ* ≤ *γπ* and - *γπ* ≤ θ ≤ *γπ* followed by the Metropolis judgment. The parameter γ and the temperature parameter T were adjusted to obtain an acceptance ratio in the Monte Carlo routine of approximately 0.5. This procedure was iterated for all residues. For a whole simulation, this routine is iterated 60000 times.

### Calculation of the contact frequency during the simulations

The contact frequency, g(i, j), between a residue pair of a given sequence (i.e., the contact probability) was calculated. In this study, whenever two Cα atoms in the i-th and j-th residues are within a 10-Å range of each other in a given conformation during a simulation, the two residues are regarded as having made a contact. A measure of high contact frequency q(μ, ν) is defined as in equation (2), where μ and ν are the μ-th and ν-th residues, respectively.
2

Here, D(m) is defined as in equations (3) and (4).
34

 expresses a residue showing a contact frequency with other residues, and this value is similar to a ϕ value [17]. We ran ten simulations for each protein, and took the average values of the simulations.

A ϕ value is an experimentally observed value defined for each residue, a value which represents the measure of each residue's involvement in native structure formation in the folding transition state [17].

In an F(μ) plot, a peak corresponds to a residue forming many inter-residue contacts. Therefore, the region around a peak is assumed to be important for folding, especially for hydrophobic collapse. Thus, F-value analysis allows us to detect the location where a hydrophobic collapse occurs. To eliminate the effects of any nonspecific contacts made by N- and C-termini, several residues, ten Gly residues, were added to the N- and C-termini.

This technique has been applied to identify the location of folding initiation sites in a protein [10–12, 16].

### Extracting local sequence tendencies

In the previous study [11], we performed the F-value analyses for two 60% identical sequences. One protein exhibited the 3α structure and the other the 4α+β structure. The sequence with the 4α+β structure was derived from that of [PDB:1PGA], and that with the 3α structure was from [PDB:1EDI]. As a result, it was observed that the 3D structure of a sequence is the 3α structure if a partial sequence around a peak in the F-value plot is similar to that of [PDB:1EDI] and vice versa. We also perform a similar treatment in the present systems. However, the differences in the sequences treated in the present study are much more subtle. Thus we introduce the following method, referred to as sequence tendency.

A sequence of GA98-1, GB98-1, GA98-2, or GB98-2 seems to be composed of combinations of some segments in the original sequences of [PDB:1PGA](GA) and [PDB:2FS1](GB). For example, residues 2–8 and 10 in above four sequences are descended from 1PGA, while residues 9 and 11–14 are from 2FS1. To clarify where the local sequences with a high similarity to [PDB:1PGA] (or [PDB:2FS1]) sequence distribute, we have carried out the following calculations.

We define a sequence tendency as follows. If a site comes from [PDB:1PGA], we score the site as 1, while if a site comes from [PDB:2FS1], we score the site as -1. After scoring every site, we smoothed them with a Gaussian kernel with h = 3.5 and plotted it as a curve.

### Gō-model simulations

A so-called Gō model is widely used to infer the folding process of a protein with a known 3D structure [18–27]. In a Gō model, only the attractive potential between contacting residues in the native protein structure is considered. In this study, a Gō model technique that we developed [27] is employed. A brief summary of this model is provided in the following text.

**Figure 12:**
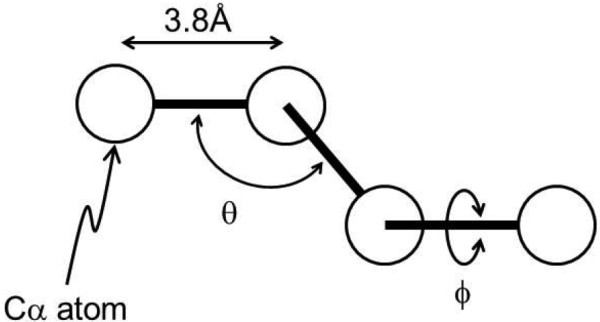
**Cα-bead model of a protein used in this study.** The bond length is fixed as 3.8 Å, and the bond and dihedral angle are symbolized as θ and ϕ, respectively.

In the present Gō-model calculations, the same beads model is again employed (Figure 12). The total energy E at a given protein conformation Γ is derived by equation (5). The total energy E of a conformation of a given protein is expressed as follows.
5

The subscript 0 refers to the native structure. The first and second terms of equation (5) denote the energies related to the virtual bond angle (θ) and torsional angle (ϕ) (Figure 12). The values of K_θ_ = 20, ,  were used in equation (5) [27]. The third term is the summation of nonlocal interaction energy between each pair of residues, i and j, that forms a contact in the native structure. Again, *r*_*ij*_ is the distance between the Cα atoms of i-th and j-th residues. In the present Gō model, a contact between the i-th and j-th amino-acid residues is defined when one of the heavy atoms in the i-th residue is within a distance of 4 Å to any heavy atoms in the j-th residue. (The 4 Å cutoff was the result of fine tuning in order that the present Gō model provides the actual folding process of a protein.) An additional "contact number" is defined in the present study, namely, the number of atom pairs with atoms closer than 4 Å from each other in a given contacting residue pair. The parameter C_ij_ in equation (5) is the contact number in the contact of i-th and j-th residues divided by the average contact number for all contacted residue pairs. In other words, C_ij_ is regarded as a scaled contact number. B_ij_ in equation (5) is defined by equations (6) and (7). This parameter takes a value between 0 and 1.
67

Here, **h**_i_ and **h**_j_ denote vectors defined by bond vectors, that is, **h**_i_ = **r**_i,i-1_+ **r**_i,i+1_ and **h**_j_ = **r**_j,j-1_+ **r**_j,j+1_, where **r**_i,i-1_ is a bond vector defined by residues i and i-1. Since Θ_i,j_ is the angle between vectors **h**_i_ and **h**_j_, this parameter can be regarded as an index of the relative orientation of i-th and j-th residues. The main determinant of the relative orientation of two residues is considered to be the relative orientation of the side chains of the two residues. Namely, the relative orientation of the side chains of the i-th and j-th residues can be expressed implicitly by Θ_i,j_. B_ij_(Θ_i,j_) is a parameter which indicates how close a given relative orientation of i-th and j-th residues is to the native one. In equation (7), *a*_Θ_ = 0.6π is used [27]. For the terminal residues, B_ij_ always equals 1, because the vector **h**i cannot be defined for them, and Θ is always less than π to prevent **h**_i_ = 0. The fourth term in equation (5) denotes repulsive interactions for non-native contacts.

To represent the structural properties of conformations during a simulation, a Q value is defined as the ratio of the native contacts in a conformation during a simulation.


Using the Q value, we can estimate the degree of native structure formation during a simulation.

### Ethical considerations

We state that this study does not include any of ethical issues like misconductive uses of individual human/animal/plant data, retrospective analyses, or clinical tools as it just includes the available data in Protein Data Bank.

## Electronic supplementary material

Additional file 1: **Additional studies for other homologous pairs are presented.** Because our study does not contain newly discovered sequences or structures, we just show the supplementary materials in this additional file. **Figures S1-S3.** show the results of sequence tendency analyses with conserved hydrophobic residues. **Figures S4-S8.** shows the results of F-value analyses for all the GA/GB pairs with conserved hydrophobic residues. (PDF 585 KB)

## Abbreviations

ADM: Average distance map
BLAST: Basic local alignment search tool
PDB: Protein data bank
IgG: Immunoglobulin G
HAS: Human serum albumin.
