# Supplementary material for: Implication of the cause of differences in 3D structures of proteins with high sequence identity based on analyses of amino acid sequences and 3D structures
Source: BMC Res Notes. 2014 Sep 18;7:654. doi: 10.1186/1756-0500-7-654 (PMC4180342; doi:10.1186/1756-0500-7-654)
Supplement: Supplementary file 1 — Additional file 1: Additional studies for other homologous pairs are presented. Because our study does not contain newly discovered sequences or structures, we just show the supplementary materials in this additional file. Figures S1-S3. show the results of sequence tendency analyses with conserved hydrophobic residues. Figures S4-S8. shows the results of F-value analyses for all the GA/GB pairs with conserved hydrophobic residues. (PDF 585 KB) [file 13104_2014_3191_MOESM1_ESM.pdf]

Results of F-value analyses for other pairs with lower sequence identities than 98%

Figures S1-S3 show the results of sequence tendency analyses with conserved hydrophobic residues. Figures S4-S8 shows the results of F-value analyses for all the GA/GB pairs with conserved hydrophobic residues.

Except for the peaks at residue 49 observed in the solid lines of Figures S4 and S5, the locations of F-value peaks seem not to change. That is, residue 49 of the GA77 and GA88 sequences would play a more important role for structural formation compared to that that of the GA98 sequence; However, because two of the peaks are similar, the pairs with lower sequence identities are considered to fold in a manner simialr to that of the current study.

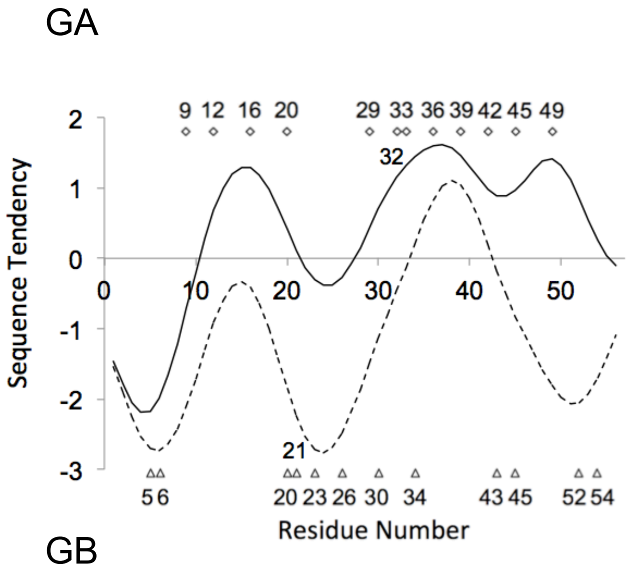

Figure S1

Figure S1 - Distribution of conserved hydrophobic residues with local sequence tendencies (GA77 and GB77)

The solid or dashed line corresponds to the sequence tendency of GA77 or GB77, respectively. The squares above the sequence tendency plot denote the conserved hydrophobic residues of 2FS1 and its homologues, while the triangles below the tendency plot denote these of 1PGA.

GA

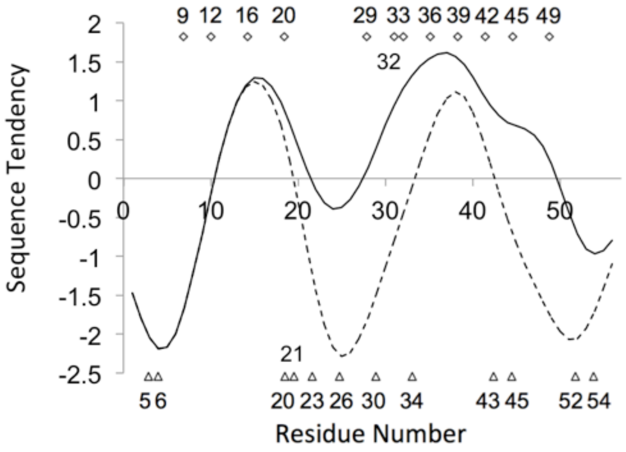

GB

Figure S2

Figure S2 - Distribution of conserved hydrophobic residues with local sequence tendencies (GA88 and GB88)

The solid or dashed line corresponds to the sequence tendency of GA88 or GB88, respectively. The squares above the sequence tendency plot denote the conserved hydrophobic residues of 2FS1 and its homologues, while the triangles below the tendency plot denote these of 1PGA.

GA

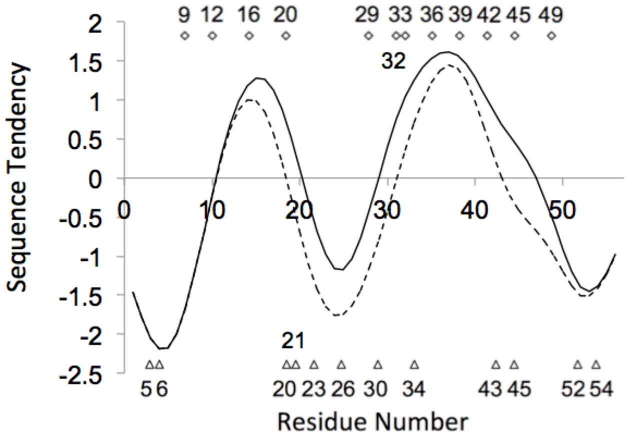

GB

Figure S3

Figure S3 - Distribution of conserved hydrophobic residues with local sequence tendencies (GA95 and GB95)

The solid or dashed line corresponds to the sequence tendency of GA95 or GB95, respectively. The squares above the sequence tendency plot denote the conserved hydrophobic residues of 2FS1 and its homologues, while the triangles below the tendency plot denote these of 1PGA.

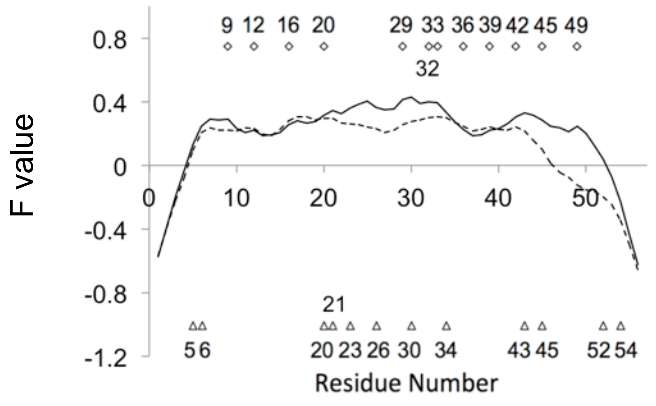

Figure S4

Figure S4 - Distribution of conserved hydrophobic residues with F-value plots (GA77 and GB77)

The solid or dashed line corresponds to the F values of GA77 or GB77, respectively. The squares above the sequence tendency plot denote the conserved hydrophobic residues of 2FS1 and its homologues, while the triangles below the tendency plot denote these of 1PGA.

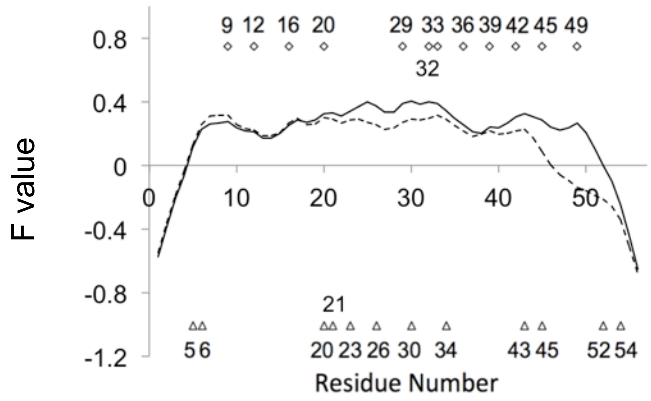

Figure S5

Figure S5 - Distribution of conserved hydrophobic residues with F-value plots (GA88 and GB88)

The solid or dashed line corresponds to the F values of GA88 or GB88, respectively. The squares above the sequence tendency plot denote the conserved hydrophobic residues of 2FS1 and its homologues, while the triangles below the tendency plot denote these of 1PGA.

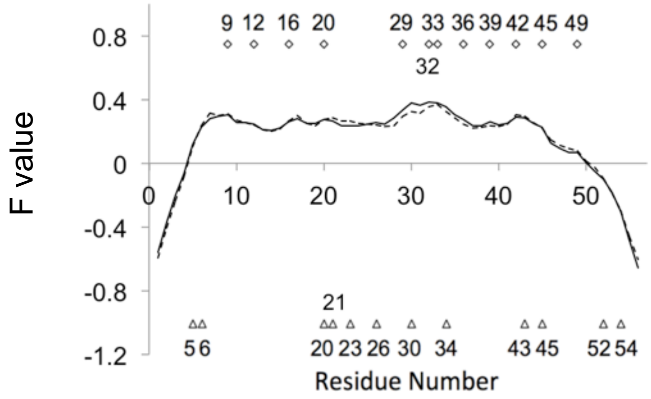

Figure S6

Figure S6 - Distribution of conserved hydrophobic residues with F-value plots (GA95 and GB95)

The solid or dashed line corresponds to the F values of GA95 or GB95, respectively. The squares above the sequence tendency plot denote the conserved hydrophobic residues of 2FS1 and its homologues, while the triangles below the tendency plot denote these of 1PGA.

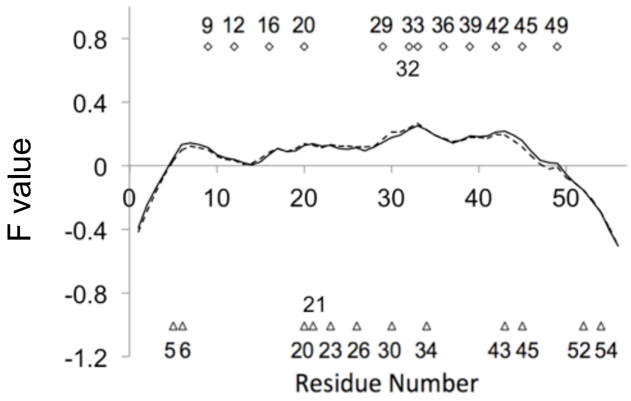

Figure S7

Figure S7 - Distribution of conserved hydrophobic residues with F-value plots (GA98-1 and GB98-1)

The solid or dashed line corresponds to the F values of GA98-1 or GB98-1, respectively. The squares above the sequence tendency plot denote the conserved hydrophobic residues of 2FS1 and its homologues, while the triangles below the tendency plot denote these of 1PGA.

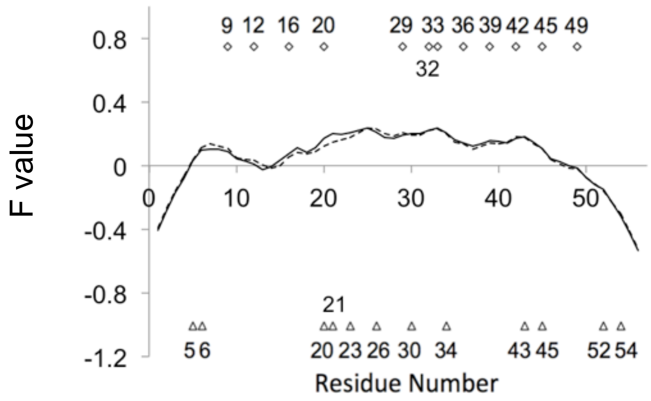

Figure S8

Figure S8 - Distribution of conserved hydrophobic residues with F-value plots (GA98-2 and GB98-2)

The solid or dashed line corresponds to the F values of GA98-2 or GB98-2, respectively. The squares above the sequence tendency plot denote the conserved hydrophobic residues of 2FS1 and its homologues, while the triangles below the tendency plot denote these of 1PGA.
